# Nanoparticle Tracking Analysis for the Enumeration and Characterization of Mineralo-Organic Nanoparticles in Feline Urine

**DOI:** 10.1371/journal.pone.0166045

**Published:** 2016-12-22

**Authors:** M. Mellema, M. Stoller, Y. Queau, S. P. Ho, T. Chi, J. A. Larsen, N. Passlack, A. J. Fascetti, C. Mohr, J. L. Westropp

**Affiliations:** 1 Department of Veterinary Surgical and Radiological Science, School of Veterinary Medicine, University of California Davis, Davis, California, United States of America; 2 Department of Urology, School of Medicine, University of California San Francisco, San Francisco, California, United States of America; 3 Royal Canin Research & Development Center, Aimargues, France; 4 Department of Preventive and Restorative Dental Sciences, School of Dentistry, University of California San Francisco, San Francisco, California, United States of America; 5 Department of Molecular Biosciences, School of Veterinary Medicine, University of California Davis, Davis, California, United States of America; 6 Institute of Animal Nutrition, Department of Veterinary Medicine, Freie Universität Berlin, Berlin, Germany; 7 Department of Pathology, Microbiology and Immunology, School of Veterinary Medicine, University of California Davis, Davis, California, United States of America; 8 Department of Veterinary Medicine and Epidemiology, School of Veterinary Medicine, University of California Davis, Davis, California, United States of America; Brandeis University, UNITED STATES

## Abstract

Urinary stone disease, particularly calcium oxalate, is common in both humans and cats. Calcifying nanoparticles (CNP) are spherical nanocrystallite material, and are composed of proteins (fetuin, albumin) and inorganic minerals. CNP are suggested to play a role in a wide array of pathologic mineralization syndromes including urolithiasis. We documented the development of a clinically relevant protocol to assess urinary CNP in 9 healthy cats consuming the same diet in a controlled environment using Nanoparticle Tracking Analysis (NTA^®^). NTA^®^ is a novel method that allows for characterization of the CNP in an efficient, accurate method that can differentiate these particles from other urinary submicron particulates. The predominant nanoscale particles in feline urine are characteristic of CNP in terms of their size, their ability to spontaneously form under suitable conditions, and the presence of an outer layer that is rich in calcium and capable of binding to hydroxyapatite binders such as alendronate and osteopontin. The expansion of this particle population can be suppressed by the addition of citrate to urine samples. Further, compounds targeting exosomal surfaces do not label these particulates. As CNP have been associated with a number of significant urologic maladies, the method described herein may prove to be a useful adjunct in evaluating lithogenesis risk in mammals.

## Introduction

Urinary tract stone formation is an ancient disease process with a recently reported prevalence of up to 20% in adults in developed nations [1]. In many parts of the world, the prevalence of urolithiasis increased during the latter portion of the 20^th^ century[2,3] and can result in numerous hospital visits, surgeries, and interventional procedures for the patient. Urolithiasis in cats is also a frequently encountered disease, and the most common urolith type in both cats and humans is calcium oxalate (CaOx) [4,5]. Relative risk factors for CaOx are similar between the two species and include male predisposition, decreased fluid intake, and highly acidified urine[6,7]. Furthermore, humans and cats are reported to have idiopathic hypercalciuria as a metabolic feature in this disease[8,9]. Pharmacologic management strategies for both species have yielded variable results[10–13]. Overall, high recurrence rates are problems in both species[14,15].

To date, studies of the physical formation of CaOx have primarily focused on processes relating to mineral or mineral-organic crystallization, nucleation, crystal growth, and aggregation. However, protein inhibitors of crystallization can bind mineral in an alternative insoluble form. These forms represent colloidal suspensions of protein-mineral aggregates that may have crystalline layers, but are not uniform crystal lattices and form by different mechanisms. These protein-mineral complexes, or calcifying nanoparticles (CNP), are spherical nanocrystallite material composed of biomineral and are 80–90 nm in diameter in their most stable (primary) form[16]; larger, less stable, secondary CNP have also been reported to occur under various conditions. CNP are composed of proteins (fetuin, albumin) and inorganic minerals[17] and are suggested to play a role in a wide array of pathologic mineralization syndromes including urolithiasis. CNP have been isolated from uroliths by several groups and are associated with the presence of renal papillary calcification (i.e. Randall’s plaques) in humans that form CaOx uroliths[18–24]. CNP formation, size distribution, and stability are all dependent on chemical factors in solution, many of which are often altered in the urine [17].

The overall small size distribution of primary as well as larger secondary CNP precludes accurate characterization with traditional flow cytometric methods. Electron microscopy has been considered the gold standard for visualization of CNP, and several other methods for detection have also been used including ELISA, immunohistochemistry, immunofluorescence, immunoblotting, and Ouchterlony immunodiffusion. However, none of these methods are suitable for detailing both the size and surface characteristics of CNP in urine samples. Nanoparticle Tracking Analysis (NTA®; Malvern Instruments) is a novel method that allows for the characterization of submicron particulates based on their Brownian movement [25–30]. NTA^®^ has been applied to exosome research, but the authors are unaware of published studies in which NTA^®^ has been used to identify, or characterize CNP. Therefore, the goals of this study were to demonstrate that feline urine CNP can be characterized, identified, and enumerated using NTA^®^. We document the development of a clinically relevant protocol to assess urinary CNP in 9 healthy cats consuming the same diet in a controlled environment.

## Materials and Methods

### Animals and urine sample collection/processing

The urine of nine (3 neutered male and 6 intact female) specific-pathogen free, healthy, adult domestic shorthair cats were used in this study. The average (range) age, body weight and body condition score of the female cats was 8.75 yr (6.5–10.75 yr), 4.32 kg (2.63–5.88 kg), and 6.75 (6.5–7.5 on a 9 point scale), respectively. The average (range) age, body weight and body condition score of the male cats was 6.54 yr (5.33–7.33 yr), 5.99 kg (5.63–6.48 kg), and 5.2 (3.5–8 on a 9 point scale), respectively. The urine samples had been saved from a separate, unrelated study, during which cats were housed at the University of California, Davis. The facility maintained room temperatures between 18–24°C and had a 14 h light/10 h dark cycle. All cats had been fed the same dry maintenance diet (Science Diet^**®**^ Adult Light; Hill’s^**®**^ Pet Nutrition Inc. Topeka, KS, USA) to maintain appropriate body condition for 4 weeks prior to urine collection by natural voiding. The experimental protocol was approved by the Institutional Animal Care and Use Committee at the University of California, Davis (Animal Welfare Assurance Number A3433-01) and the Royal Canin Ethics committee complied with the recommendations of the 2011 National Research Council Guide for the Care and Use of Laboratory Animals (The National Academies Press, Washington DC).

Voided urine samples were collected from the cats during individual housing and then placed into sterile centrifuge tubes and maintained at 4 deg C until analysis. Urine was centrifuged at 1500 xG for 5 minutes then sterile filtered through a 0.22 um syringe filter prior to storage. Aliquots were stored at 4, -20, and -80 deg C. Refrigerated samples were split into two with one fraction having no additives and another containing 109 mmol/L of the dihydrate form of trisodium citrate Na_3_C_6_H_5_O_7_ 2H_2_0 as a calcium-chelating agent.

### CNP Stock Solution Standards-In vitro generation

Positive control suspensions consisted of CNP stock solutions generated using previously published protocols[16]. In brief, CNP can be generated on the benchtop by the careful mixture of three stock solutions: Stock solution 1 was a NaCl solution: 140 mM NaCl, Stock solution 2 was a calcium solution: 40 mM CaCl_2_ +100 mM Hepes +140 mM NaCl pH-adjusted with 10 M NaOH to 7.40 at 37°C; Stock solution 3 was a phosphate solution: 19.44 mM Na_2_HPO_4_ + 4.56 mM NaH_2_PO4 + 100 mM Hepes + 140 mM NaCl pH-adjusted with 10 M NaOH to 7.40 at 37°C. Calcified nanoparticle formation was induced by mixing reagents in the following sequence at 37°C: (1) NaCl solution: 20 volumes, (2) fetuin-A [0.1%]: 80 volumes, (3) shaking for 1 minute, (4) phosphate solution: 50 volumes, (5) shaking for 1 minute, and (6) calcium solution: 50 volumes and shaking for 1 minute. All chemicals, including lyophilized fetuin-A derived from fetal calf serum, were purchased from Sigma-Aldrich (St. Louis, MO, USA).

### Nanoparticle Tracking Analysis

Calcified nanoparticles in the whole urine samples and *in vitro* generated CNP suspensions were analyzed using the NanoSight LM10HS-48814TS instrument (Malvern Instruments, Worcestershire, UK) with a 488 nm wavelength laser, fluorescent filter (505 nm long pass), and both temperature control and syringe pump modules. The analysis settings were optimized and kept constant between samples, and each video was analyzed to give the mean, mode, median, and estimated concentration for each particle size. Advanced script control options were used for each analysis which encompassed an 80 uL syringe pump driven chamber-priming interval, a 30 second pause to minimize vibration artifact, three 60 second video capture periods with constant syringe pump-driven sample delivery, and automated laser and pump shutdown after video acquisition. Samples were analyzed with fluorescent filter in-line only when fluorophores had been applied to the sample for surface labeling. Following published methods for NTA characterization of urine exosomes[31] and initial pilot studies comparing whole urine samples and 1:1000 dilution, all experiments were carried out at a 1:1000 dilution, yielding particle concentrations in the region of 1 x10^8^ particles/ml in accordance with the manufacturer’s recommendations. All samples were analyzed in triplicate.

Fluorescent labeling was performed with four distinct strategies. Firstly, Exo-FITC (SBI; Mountain View, CA) was employed to quantitate urine exosomes amongst our particle populations. Exosomes are a type of extracellular microvesicle (EMV) with a diameter range (30–200 nm) that overlaps with the size range of CNP (80–500 nm). Exosomes are generally defined as being endocytic in origin, produced by the inward budding of multivesicular bodies (MVBs). They are released from the cell into the microenvironment following fusion of MVBs with the plasma membrane. Exo-FITC is a commercially available universal and reversible exosome label that relies on the unique surface characteristics of exosomes to achieve binding. Exosome positive controls, derived from cultured human cells, were generously donated by the Nolta Lab (Center for Regenerative Cures, UC Davis School of Medicine, Davis, CA, USA). Secondly, Fluo-4 (CNP) and Fluo-4-AM (exosomal controls) were used to selectively label particulates with high calcium content as has been previously reported by others[32]. Labeled calcium indicators are molecules that exhibit an increase in fluorescence upon binding Ca^2+^. Fluo-3 has long been used to image the spatial dynamics of Ca^2+^ signaling in cell biology. Fluo-4 is an analog of Fluo-3 with the two chlorine substituents replaced by fluorines, which results in increased fluorescence excitation at 488nm and consequently higher fluorescence signal levels. Lastly, two different molecules that selectively and specifically bind to calcium-phosphorus surfaces (alendronate, osteopontin) were conjugated to a fluorophor. Fluorophor labeling was performed with a commercially available kit (Lightning Link® Rapid Dylight® 488 conjugation kit; Innova Bioscience, Babraham, UK) designed for this purpose according to the manufacturer’s recommendations. Alendronate sodium hydrate was obtained from Cayman Chemicals (Ann Arbor, MI, USA) and recombinant mouse osteopontin was obtained from R&D Systems (Minneapolis, MN, USA).

All samples were diluted in 0.9% saline (Baxter) that had been sequentially filtered thru 0.8, 0.2, 0.1, and 0.02 syringe filters (Whatman) to remove background particulate matter. Each sample was analyzed under two conditions. On the first run through the NTA the fluorescent long-pass filter was left out of the path to the camera. Thus, one first captured the characteristics of all the suspended particles in the 30–2000 nm size range. Next, the long-pass filter was placed in line and the characteristics of only the fluoro-labeled sub-population (i.e. CNP) was captured. All plasticware was rinsed with this same saline prior to usage. NTA was used to confirm the absence of detectable particulates after each saline batch was generated. NTA calibration was performed monthly according to manufacturer’s recommendations.

All data files were compiled and maintained using an open-source office suite (LibreOffice5). Because transmission electron microscopy (TEM) is considered the gold standard for the identification of CNP, TEM was performed on the *in vitro* synthesized CNP stock solutions as described above. TEM studies were also performed to identify CNP formed *in vivo* in the urine of healthy, colony cats. In each case 5 microliters of samples were applied to a coated copper mesh and excess fluid wicked away. After complete air-drying, each prepared mesh was inspected with electron microscopy at 80KV.

## Results

### Submicron particulates in normal feline urine

**Fig 1:**
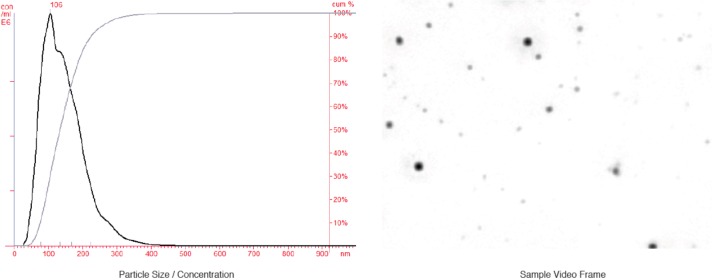
Representative histogram showing size distribution and cumulative percentage (left) of submicron particulate matter in urine from healthy cats. A representative frame from the captured video analyzed by NTA is shown as well (right).

Using laser light-scatter mode, the NTA consistently identified a population of submicron particles within the CNP size range. Summary results from the 9 cats are presented in Table 1 and a representative histogram is shown in Fig 1.

**Table 1:** Characteristic of submicron particulates in healthy cat urine (n = 9).

	Diameter	Diameter	Diameter	Diameter	Diameter	Concentration
	(nm)	(nm)	(nm)	(nm)	(nm)	(10^10^ per mL)
	mean	mode	10th	50th	90th	
Mean	165.22	68.78	51.56	114.11	340.67	2.75
SE	9.90	8.44	4.32	11.44	31.72	0.24

Mean and mode as well as values for the 10^th^, 50^th^, and 90^th^ percentile are shown. In addition, concentration in particles (x10^10^) per mL of undiluted urine is presented.

As shown in Table 1, the mode of the diameter measurements is typical for what has been reported for primary CNP by other modalities such as dynamic light scattering[16] and the size range (10^th^ to 90^th^ percentile) lies well within the reported size range of CNP [33]. The histogram is suggestive of a sample in which a subset of CNP has undergone transition to larger forms (secondary CNP) and the size of the larger forms is consistent with prior reports[16].

As the size of this particle population is also consistent with exosomes, it is important to exclude these extracellular vesicles from the analysis. Staining of feline urine samples with Exo-FITC® resulted in no detectable particle population using NTA fluorescence mode. As a positive control, exosomes derived from cultured human cells and concentrated with precipitation methods (ExoQuick®) were readily identified at concentrations as low as 1 x10^7^ particles per milliliter (data not shown). Moreover, the number of presumptive CNP identified in light-scatter mode is orders of magnitude larger than has been previously reported for urinary exosomes in humans[30]. Lastly, the refractive index (RI) of exosomes in human urine is reported to be on the order of 1.37 [27] whereas the particles identified in feline urine have an RI that is 3–10 times greater.

**Fig 2:**
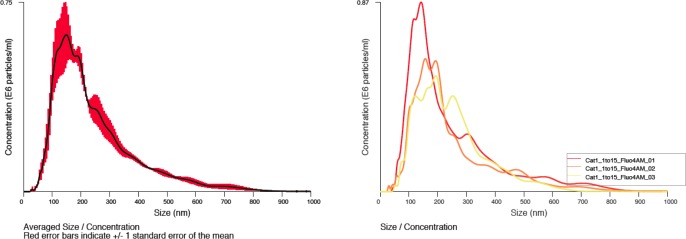
Fluo-4-AM positive particulate matter from a healthy cat. A representative histogram showing averaged sizing and relative abundance data (with SE in red) and individual analyses from the triplicate assessment (right) cumulative percentage (left) of submicron particulate matter in healthy feline urine.

To determine whether the particle populations observed contained a calcium-rich outer shell as is typical of CNP, we repeated NTA analysis in the presence of Fluo-4-AM at a final concentration of 1 mM. In each sample, the predominant particulate population was strongly positive for Fluo-4-AM indicating calcium content well in excess of background levels. A representative summary histogram (left) and individual analyses from the triplicate assessment for cat #1 is shown in Fig 2.

**Fig 3:**
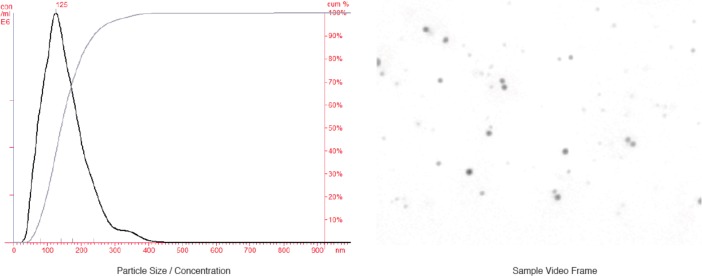
Representative histogram showing size distribution and cumulative percentage (left) of submicron particulate matter from urine obtained from a healthy cat. After a 4 hr incubation at 37 deg C. A representative frame from the captured video analyzed by NTA is shown as well (right).

One of the defining features of CNP has been their ability to increase in number under suitable, cell-free conditions [34]. This spontaneous formation in cell-free culture media was one of the original features that led to them being named nanobacteria and presumed to be living organisms. In cell culture media, this expansion in number occurs slowly taking a matter of weeks. In previous in vitro studies, CNP numbers (and average size) have been shown to increase substantially within a matter of hours. For each of our 9 urine samples, analysis was repeated after a 4 hr incubation at 37 deg C. The results of this analysis are shown in Table 2 and a representative data set is displayed in Fig 3. Particulate concentrations increased approximately 4-fold during this incubation period and both the median as well as the mode of the particle diameters increased suggesting both a substantial number of new particles forming as well as an increase in their size. This increase in both particle number and size was inhibited by the addition of a calcium chelator (citrate) to the samples (data not shown).

**Table 2:** Characteristic of submicron particulates in healthy cat urine (n = 9) after a 4 hr incubation at 37 deg C.

	Diameter	Diameter	Diameter	Diameter	Diameter	Concentration
	(nm)	(nm)	(nm)	(nm)	(nm)	(10^10^ per mL)
	mode	mode	10th	50th	90th	
Mean	142.11	114.44	71.89	131.22	222.78	10.39
SE	9.90	8.44	4.32	11.44	31.72	0.98

Mean and mode as well as values for the 10^th^, 50^th^, and 90^th^ percentile are shown. In addition, concentration in particles (x10^10^) per mL of undiluted urine is presented.

### Specific binding of fluorophor-conjugated alendronate and osteopontin

**Fig 4:**
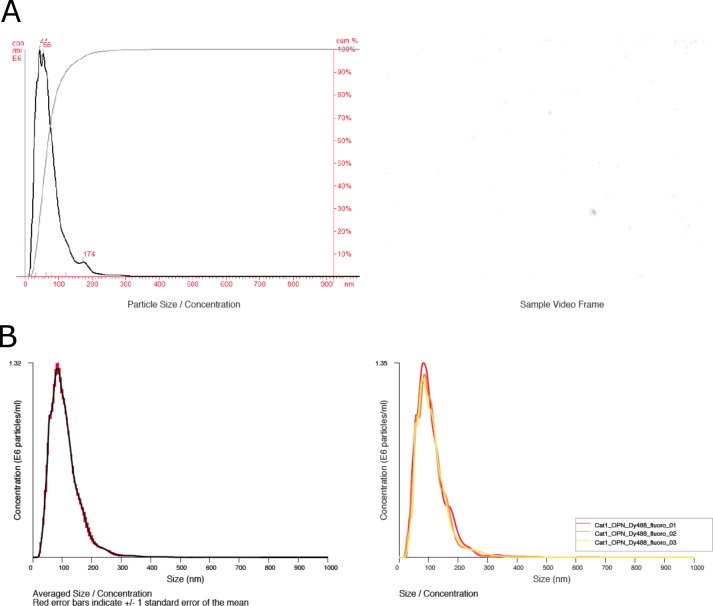
**A**: Representative histogram showing size distribution and cumulative percentage (left) of submicron particulate matter in healthy feline urine after labeling with DyLight 488 conjugated alendronate. A representative frame from the captured video analyzed by NTA is shown as well (right). Note the strongly preferential binding to primary CNP that lack an outer layer of protein or mucus. **B**: Representative histogram showing averaged sizing and relative abundance data (left; SE in red) and individual analyses from the assessment (right) of this representative sample of healthy feline urine after labeling with DyLight 488 conjugated osteopontin. Note the strongly preferential binding to primary naturally-occurring CNP that lack an outer layer of protein or mucus.

**Fig 5:**
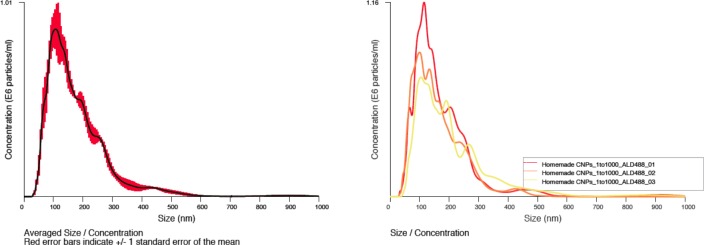
DyLight488-conjugated alendronate binding of CNP generated *in vitro*. A representative histogram showing averaged sizing and relative abundance data (left; SE in red) and individual analyses from the assessment (right) of this representative sample in triplicate. Note that CNP generated *in vitro* do not acquire the protein/mucus coating known to occur *in vivo* and the larger forms retain the ability to bind to alendronate.

**Fig 6:**
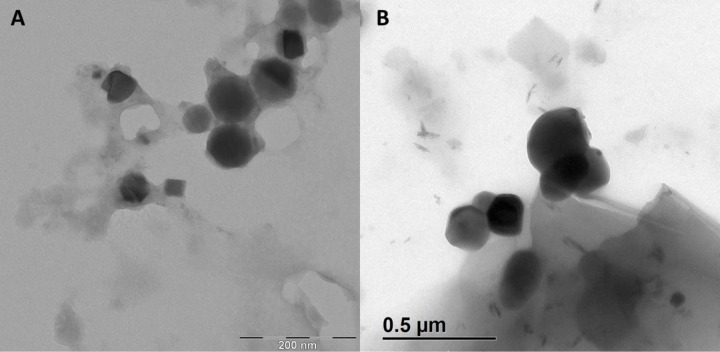
Transmission electron microscopy (TEM) images of calcifying nanoparticles (CNP). (A) CNP synthesized de novo in the laboratory using supersatured solutions of calcium and phosphorus and a solution of bovine fetuin A (see detailed description in the methodology section). (B) CNP identified in the urine of healthy, colony cats. In each case 5 microliters of samples were applied to a coated copper mesh and excess fluid wicked away. After complete air-drying, each prepared mesh was evaluated with electron microscopy at 80KV.

Primary CNP consist of an inner cavity surrounded by a hydroxyapatite outer shell. Larger secondary CNP may increase in size following the acquisition of an additional outer layer of protein (e.g. fetuin-a) or mucus (which can be detected by staining with 2% uranyl acetate)[23]. Thus, primary CNP, but rarely secondary CNP should specifically bind to agents with affinity for hydroxyapatite surfaces. To exploit this property we fluorescently labeled two such agents (alendronate, a bisphosphonate compound, and osteopontin, a hydroxyapatite-binding protein expressed in bone) and treated all feline urine samples with these compounds prior to repeating NTA in fluorescence mode. With both agents, specific binding to the nanoparticulates present in feline urine was observed (Fig 4A and 4B) while no such binding was observed with polystyrene control beads (100 nm mean diameter). Preferential binding to primary CNP is detected by the shift in the histogram to the left (smaller sized population). CNP generated in vitro cannot acquire this mucus layer and larger CNP retain the ability to bind alendronate (Fig 5). Transmission electron microscopy was used to characterize the urine particulates in parallel with NTA evaluation (Fig 6).

## Discussion

Submicron particulate matter appears to be relatively abundant in feline urine as evidenced by NTA of the samples we analyzed. In our nine cats with no known history of urolithiasis, the average concentration of submicron particulates was 2.7 +/- 0.21 x10^10^ particles per milliliter of urine. Several lines of evidence suggest strongly that this particulate matter is largely mineralo-organic and not exosomal in nature. While urine exosomes are quite numerous and represent an important potential source of novel biomarkers, exosomes would generally require specific induction (e.g. with antidiuretic hormone) or post-acquisition enrichment to reach the concentrations in mammalian urine observed herein.

We have demonstrated that the submicron particulates present in feline urine fall within established ranges for CNP and have the characteristic structural features of CNP in TEM images. In our feline samples the 10^th^ to the 90^th^ percentile of particle diameter averaged at 54–362 nm, which is entirely consistent with CNP sizes established by other modalities. However, NTA has several advantages over other reported methods for CNP analysis (e.g. dynamic light scattering among others) and can be coupled with fluorescent labeling to allow for immunophenotyping of submicron particle sub-populations. CNP are mineralo-organic colloidally suspended particles that play a role in both physiologic mineral homeostasis as well as in diseases characterized by ectopic extra-skeletal mineralization. Of particular importance is the potential role of CNP in urinary tract lithogenesis. CNP have been isolated from uroliths, Randall’s plaques, and bladder mucosa in encrusted urinary bladder cystitis[18–24]. NTA can characterize particle populations ranging from 30–1000 nm in size and requires as little as one microliter of biological fluid sample. This minute sample size requirement may allow for investigations to be performed in other urolithiasis models wherein urine volumes are exceedingly small such as *Drosophila sp*.[35,36].

Size distribution alone is insufficient to establish these nanoparticulates as CNP. We have demonstrated that under sterile, cell-free conditions these particulates can increase in number nearly 4-fold which is consistent with the behavior described for CNP generated *in vitro* by others[16]. Further, due to the overlap of the CNP size range with that for exosomes it is essential to distinguish these two nanoparticulate populations. While the refractive index (RI) of the submicron particulates in feline urine is much greater than is reported for urinary exosomes, the investigators deemed that this alone was insufficient to exclude exosomes from the analysis. For this reason, NTA following staining with a specific and reversible exosome-binding fluorophore (Exo-FITC®) was performed as well. At the urine dilutions used in this study, Exo-FITC staining particles were below the detection limit for NTA. Human exosomal preparations were detectable at concentrations as low as 1x10^7^ particles/mL. Given the high RI of the particles of interest in our samples and the lack of Exo-FITC® binding, the authors concluded that exosomes make up only a very minor portion of the particle populations in our samples. Moreover, the submicron particle identified with electron microscopy lack the characteristic cup shape of exosomes. Further, the increase in size was inhibited by the addition of the chelator citrate, which has also been reported to help inhibit urinary crystallite deposition and CaOx stone formation[37].

Fluo-4 is a calcium-activated fluorophore that has been used previously to detect nanoscale calcium-containing crystals[32]. In the present investigation, we employed this agent to confirm that the predominant submicron particles present in feline urine have a calcium-rich outer layer which is strongly supportive of their being CNP. Application of this stain to other particles notably polystyrene beads) failed to produce particulates detectable by fluorescence though they were identifiable by laser light scatter.

The outer layer of primary CNP is made up predominately of calcium and phosphorus in the form of hydroxyapatite (HA). We employed a labeling strategy herein where a fluorophore (DyLight488) was conjugated to two compounds that bind specifically to HA, but via different binding motifs. When these labeled compounds were applied to feline urine samples prior to NTA, they efficiently and specifically labeled primary CNP. Primary CNP formed *in vivo* lack the external coating of protein and mucus that is identified on larger secondary CNP. The expectation that our labeled products would preferentially bind to the smaller particulates in our samples was born out by the empiric data. Similarly, when CNP generated *in vitro* were subjected to this same analysis no preferential binding to smaller CNP was observed. CNP generated in vitro can transition to secondary CNP, but lack the protein/mucus coating that can reduce access to HA surfaces by alendronate and osteopontin.

The limitations of this study bear mentioning. The sample size is modest and limited to a single mammalian species, which limits the broader applicability of our findings. The authors elected to not culture the presumptive CNP although this technique has been widely used by others. While culture techniques offer a means of propagating CNP, they offer no additional insights into the particle characteristics.

In summary, we have demonstrated that NTA can provide a detailed accounting of the submicron particulate matter in mammalian urine. This method requires as little as 1–5 microliters of urine and is thus suitable for use with even the smallest animal models of urolithiasis (e.g. fruit flies). The predominant nanoscale particulates in feline urine have the characteristics of CNP based on their size, their ability to spontaneously form under suitable condition, and the presence of an outer layer that is rich in calcium and capable of binding to hydroxyapatite binders such as alendronate and osteopontin. As CNP have been associated with a number of significant urologic maladies, including nephrolithiasis [38], the method described herein may prove to be a useful adjunct in evaluating lithogenesis risk in mammals. We propose that urinary CNP indices may prove to be a useful adjunct in the evaluation of lithogenic risk and future studies evaluating CNP formation in cats and humans with urolithiasis are warranted
